# Excision of the Tonsils

**Published:** 1828-04

**Authors:** A. H. Hosack


### II. ANALYTICAL.

4. *Excision of the Tonsils.* In the second number of the American Journal of the Medical Sciences, Dr. A. H. Hosack, of New-York, has published a paper strongly advising excision of the tonsils, in preference to the ligature. The operation, he says, gives little pain, can be performed in an instant, never excites convulsions, and originates less inflammation and fever than the ligature; even when applied on the improved method of Dr. Physick, who himself, as we are told in a note by Dr. Horner, "decidedly prefers excision." Dr. Hosack has lately performed the operation, successfully, in six cases, one of which is cited. His instructions are briefly these:—

"Nothing more is necessary than to pass a hook through the body of the enlarged gland, and to raise it from its bed between the arches of the palate, then to pass a probe-pointed bistoury under its base, and with one stroke to sever it from its connexion: the hæmorrhage never exceeds two ounces, and is always to be arrested by a draught of cold water." p. 312.

